# Fungal Endophytes Exert Positive Effects on *Colobanthus quitensis* Under Water Stress but Neutral Under a Projected Climate Change Scenario in Antarctica

**DOI:** 10.3389/fmicb.2020.00264

**Published:** 2020-02-28

**Authors:** Rasme Hereme, Samuel Morales-Navarro, Gabriel Ballesteros, Andrea Barrera, Patricio Ramos, Pedro E. Gundel, Marco A. Molina-Montenegro

**Affiliations:** ^1^Instituto de Ciencias Biológicas, Universidad de Talca, Talca, Chile; ^2^Bachillerato en Ciencias, Facultad de Ciencias, Universidad Santo Tomás, Talca, Chile; ^3^Núcleo Científico Multidisciplinario-DI, Universidad de Talca, Talca, Chile; ^4^IFEVA, CONICET, Facultad de Agronomía, Universidad de Buenos Aires, Buenos Aires, Argentina; ^5^Centro de Estudios Avanzados en Zonas Áridas, Universidad Católica del Norte, Antofagasta, Chile; ^6^Centro de Investigación en Estudios Avanzados del Maule, Universidad Católica del Maule, Talca, Chile

**Keywords:** functional symbiosis, Antarctica, climate change, *Colobanthus quitensis*, osmoprotective molecules, water stress, abscisic acid

## Abstract

Functional symbiosis is considered one of the successful mechanisms by which plants that inhabit extreme environment improve their ability to tolerate different types of stress. One of the most conspicuous type of symbiosis is the endophyticism. This interaction has been noted to play a role in the adaptation of the native vascular plant *Colobanthus quitensis* to the stressful environments of Antarctica, characterized by low temperatures and extreme aridity. Projections of climate change for this ecosystem indicate that abiotic conditions will be less limiting due to an increase in temperature and water availability in the soil. Due to this decrease in stress induced by the climate change, it has been suggested that the positive role of fungal endophytes on performance of *C. quitensis* plants would decrease. In this study, we evaluated the role of endophytic fungi on osmoprotective molecules (sugar production, proline, oxidative stress) and gene expression (*CqNCED1*, *CqABCG25*, and *CqRD22*) as well as physiological traits (stomatal opening, net photosynthesis, and stomatal conductance) in individuals of *C. quitensis*. Individual plants of *C. quitensis* with (E+) and without (E−) endophytic fungi were exposed to simulated conditions of increased water availability (W+), having the current limiting water condition (W−) in Antarctica as control. The results reveal an endophyte-mediated lower oxidative stress, higher production of sugars and proline in plants. In addition, E+ plants showed differential expressions in genes related with drought stress response, which was more evident in W− than in W+. These parameters corresponded with increased physiological mechanisms such as higher net photosynthesis, stomatal opening and conductance under presence of endophytes (E+) as well as the projected water condition (W+) for Antarctica. These results suggest that the presence of fungal endophytes plays a positive role in favoring tolerance to drought in *C. quitensis*. However, this positive role would be diminished if the stress factor is relaxed, suggesting that the role of endophytes could be less important under a future scenario of climate change in Antarctica with higher soil water availability.

## Introduction

Drought is one of the most studied abiotic stress factors with significant and negative ecological and agronomic impacts (Zhao and Running, 2010; Feller and Vaseva, 2014). The stress occurs when soil water availability is reduced to critical levels that stop growth and normal functioning of plants (Ramakrishna and Ravishankar, 2011). In response to drought, however, plants can display a suite of physiological, biochemical, and morphological adjustments to diminish the negative impact of the stress on fitness (Potters et al., 2007; Atkinson and Urwin, 2012; Osakabe et al., 2013; Tátrai et al., 2016). Accumulating recent research suggests that these phenotypic adjustments that allow plants to face stressful situations may be positively modulated by symbiotic microorganisms (Dupont et al., 2015; Wani et al., 2015). Here, we evaluated the role of fungal endophytes in the capacity of an Antarctic plant species to cope with current and projected scenarios of water availability.

Among the physiological responses that plants deploy to face stress by water deficit is an increase of osmoregulatory molecules which stabilize cell membranes and maintain cell turgor (Fang and Xiong, 2014). For example, proline and soluble sugars are osmoprotective molecules, which accumulate in response to different factors of environmental stress such as drought (Moustakas et al., 2011), and reduce the damage caused by oxidative stress (Labudda, 2013). At the hormonal level, one of the most studied phytohormones in response to drought stress is the abscisic acid (ABA). It is directly involved in the induction of stomatal closure via regulation of guard cells (Lim et al., 2015). It has been noted that an overexpression of *NCED1*, (9-cis-epoxycarotenoid dioxygenase), a key enzyme in the synthesis of ABA (Iuchi et al., 2001), promotes a greater accumulation of the hormone. Likewise, the overexpression of the ABA transporter, ATP-binding cassette G25 (*ABCG25*), increases the stress tolerance to drought as it maintains a greater efficiency in water usage (Kuromori et al., 2016). In addition, in the enzymatic cascade of stress responses induced by water deficit, there is an upregulation of ABA dependent-pathway genes (e.g., *RD22*) which are sensitive to dehydration (Shinozaki and Yamaguchi-Shinozaki, 2007). From the mechanistic point of view, it is important to identify the different steps in the signaling cascade that regulate the level of ABA and turn out in a continuous adjustment of plants to variations in water availability.

The endophyticism, defined as the mutualistic association of plants with microorganisms—either fungi or bacteria—that live inside host tissues without causing any harm, is one the most conspicuous symbiotic associations occurring in extreme environments (Kusari et al., 2012). Endophytes are known to perform or modulate various essential functions in plants directly affecting growth, development and tolerance to stress conditions (Choudhary, 2012; Wani et al., 2015; Molina-Montenegro et al., 2016; Acuña-Rodríguez et al., 2019). Among the mechanisms deployed by endophytes are the production of various chemical metabolites (e.g., plant growth hormones, secondary compounds), as well as the modulation of gene expression and other secondary metabolic pathways of the host (Long et al., 2008; Bacon and White, 2015; Deepika et al., 2016; Ramos et al., 2018). Thus, comparative studies regarding responses and profiles of differential expressions of genes in plants with and without endophytes can be useful to unravel the possible mechanisms involved in the homeostasis maintenance. However, there are very few studies that evaluating the responses at molecular and physiological levels in plants exposed to drought establish a link with the differential gene expression mediated by the root-fungal endophytes.

Successful functional symbiotic relationships have been studied and certain effects have been identified that modulated by fungal endophytes, can explain the better performance of endophyte-symbiotic plants compared to endophyte-free plants. For example, endophyte inoculated plants can display higher photosynthetic efficiency as well as a lower production of reactive oxygen species (ROS), which is suggested to be controlled by an endophyte-mediated improvement of the plant antioxidant system (Obledo et al., 2003; Hamilton et al., 2012; Azad and Kaminskyj, 2015). At the molecular level, Dupont et al. (2015) found that the inoculation of *Lolium perenne* plants with the leaf fungal endophyte *Epichloe festucae* (strain Fl1), caused changes in the expression of more than a third of the host genes, favoring secondary metabolism. In harsh environments which impose severe limitations for growth, the establishment of functional symbioses with microorganisms can play a fundamental role in adaptation of plants due to a greater accumulation of solutes, reduced foliar conductance, decreased transpiration, or the formation of thicker cuticles (Malinowski and Belesky, 2000; Hamilton et al., 2012; Timmusk et al., 2014; Lata et al., 2018).

The Antarctic ecosystem stands out for being one of the most severe environments on earth, characterized mainly by very low temperatures, strong winds, and extreme aridity, since it receives scarce liquid precipitation and the water is found primarily in the form of ice (Robinson et al., 2003; INACH, 2006; Elnitsky et al., 2008). Despite these adverse climatic conditions, two vascular plant species are considered natives to the continent: *Deschampsia antarctica* and *Colobanthus quitensis* (Parnikoza et al., 2007). Although many studies have evaluated the mechanisms that allow *D. antarctica* to persist in Antarctica, the presence of *C. quitensis* is still considered an enigma (*sensu* Smith, 1997). In fact, it has not yet been possible to establish the underlying adaptive mechanisms by which *C. quitensis* survives to such extreme conditions. The distribution range of *C. quitensis* in Antarctica is dictated by low temperature and water availability (Hughes, 2000; Cannone et al., 2016). However, these limitations are changing due to the global warming. For the Antarctic continent, the increase in mean temperature registered globally is determining the existence of new ice-free areas and increased water availability in the soil (Lee et al., 2017). This climatic tendency and the biophysical consequences are expected to favor the expansion range of vascular plant populations (Smith, 1994; Cannone et al., 2016; Acuña-Rodríguez et al., 2017). Different studies have focused on traits such as xerophytic anatomical characteristics, capacity to maintain positive net photosynthesis at low temperature, and tolerance to freezing temperature, excess radiation and water shortage (Bravo and Griffith, 2005; Molina-Montenegro et al., 2012; Acuña-Rodríguez et al., 2017; Ramos et al., 2018). Although some other studies have evaluated the role that functional symbiosis plays in the adaptation of Antarctic plants to the environment (e.g., Oses and Molina-Montenegro, 2014; Torres-Díaz et al., 2016), there is no work establishing a direct link between the endophyte-modulated gene expression, physiological performance, and individual fitness.

Here, we assessed the effects of root fungal endophytes on the performance of the Antarctic plant *C. quitensis* exposed to different water conditions. Based on the conceptual framework and background previously exposed, we performed the following predictions: (*i*) fungal-endophytes will increase tolerance to water stress in *C. quitensis* plants through the accumulation of osmoprotective molecules and maintaining higher gas exchange, by modulating the expression of genes related to the synthesis pathway of ABA, and (*ii*) this positive effect of endophytic fungi on plant performance will be less relevant under a projected climate change scenario characterized by environmental conditions that are less limiting for growth. Under this latter condition, the role of root fungal endophytes for the plant ecological fitness would turn out to be less important.

## Materials and Methods

### Study Site and Experimental Design

Fifty individuals of *C. quitensis* were collected in three sampling sites: Punta Arenas, in the Sub-Antarctic zone (53°51′S), King George Island, in the South Shetland Islands (62°09′S), and on the Lagotellerie island, in the Antarctic Peninsula (67°53′S). The plants from each sampling point were collected within the framework of the Antarctic Scientific Expedition, during the 2017–18 growing season. Each individual plant was collected along with the soil around the roots and placed in a plastic box (120 × 70 × 50 cm). All the plants were well-irrigated and maintained under natural light and temperature conditions until they were brought to the Institute of Biological Sciences of the University of Talca, Talca, Chile (35°25′S).

Once the plants collected in the field reached a size of 2 cm in diameter, half of them were subjected to a treatment with antifungal and antibiotics to obtain individuals without endophytes (E−). The other half was only given antibiotics to eliminate the effect of bacteria; thus, only the effect of fungi endophytes was evaluated in plants (E+) (for more details, see Torres-Díaz et al., 2016; Gallardo-Cerda et al., 2018). The experiment consisted of evaluating the biochemical, molecular, and functional responses of individuals under conditions of current water availability (W−) recorded in the South Shetland Islands compared to projected climate change conditions (W+) for the same area, i.e., 25% more water availability (see Torres-Díaz et al., 2016). For each water condition (current and projected), the effect of the presence of endophyte fungi (E+) was evaluated and compared to plants without endophytic fungi (E−). To do so, the individuals were transplanted in pots of 50 cc filled with a mix of substrates (soil from study site-sand-perlite) and divided into four groups corresponding to four treatments (*n* = 25 plants per group). Plants were put inside of different growth chambers with controlled conditions of light (350 mom^–2^s^–1^), relative humidity (75%), and temperature (4°C). Plants from different treatments were arranged in each growth chambers in order to avoid any effect of chambers.

**FIGURE 1:**
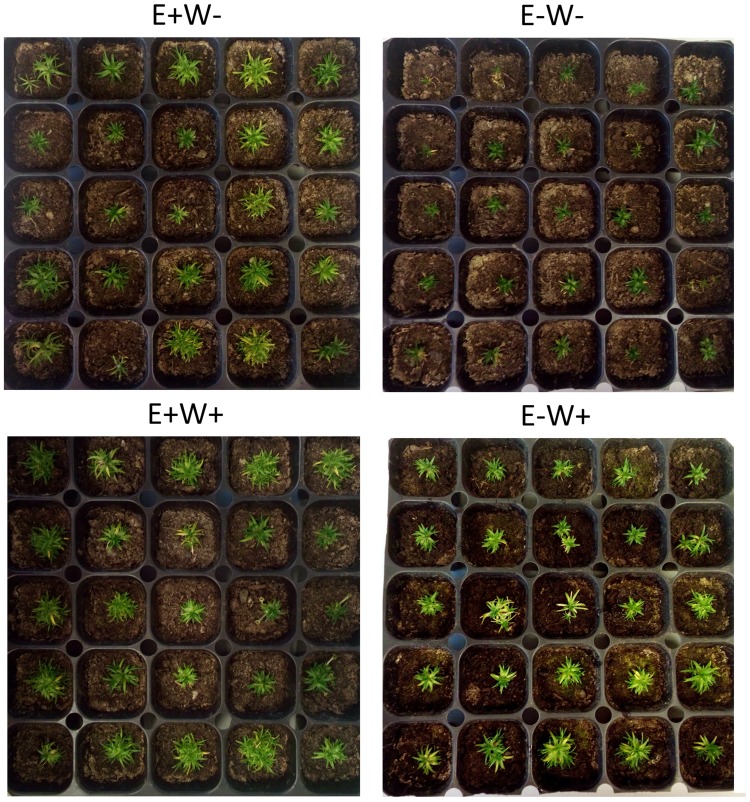
Visual aspect of *Colobanthus quitensis* plants growing with (E+) and without (E−) endophytic fungi under water condition currently found in Antarctica (W−) and under projected one according to climate change forecasts (W+).

The treatments consisted of: (*i*) presence of endophytes and current water availability (E + W), (*ii*) presence of endophytes and projected water availability (E + W +), (*iii*) absence of endophytes and current water availability (E−W), and (*iv*) absence of endophytes and projected water availability (E-W +) (see Figure 1). The current water availability in the study area of Antarctica as well as the amount of water added to estimate water availability projected by climate change was obtained from a previous study performed by our research group (see Molina-Montenegro et al., 2016). In short, to evaluate the effect of differences in water availability between the current and projected condition, individuals were watered at the beginning of the experiment with 4 and 7 ml, respectively. These values were calculated based on bibliographic data and a water potential experiment performed in the same containers and climatic conditions where the experiment would be carried out. The treatments were maintained with a photoperiod and mean Antarctic temperature during the summer season (21/3 day/night and 4°C, respectively).

In order to know the main species of endophytes inhabiting in *C. quitensis* root, we conducted a molecular identification. To do this, we amplified the ITS region of DNA that was extracted from mycelia in active growing using the E.Z.N.A. fungal DNA MiniKit (Omega-Biotek). After sequencing, the fragments of forward and reverse sequences were edited using Geneious v5.4 software (Drummond et al., 2011). The sequence of each endophyte isolated was analyzed with MegaBLAST (Basic Local Alignment Search Tool)^1^ in order to determine the percentage of maximal identity and total scores with the sequences of that global database. Overall, we found five species of endophytes with more than 1% of frequency of occurrence in root samples (*n* = 50): *Penicillium chrysogenum* (62%), *Penicillium brevicompactum* (27%), *Alternaria* sp. (6%), *Phaeosphaeria* sp. (3%), and *Eupenicillium osmophilum* (2%).

### Determination of Biochemical Responses

#### Synthesis of Osmoprotective Molecules and Membrane Damage

To evaluate whether the presence of endophytes regulates some parameters related to the potential mechanisms of drought tolerance, the synthesis of proline, total soluble sugars (TSS), and lipid peroxidation were examined in a temporal kinetic, taking foliar tissue samples of 12 individuals per treatment at 0, 12, 36, 48, and 120 h.

For the analysis of TSS, leaf tissue was suspended in ethanol (86%) for 24 h and subsequently centrifuged at 1200 *g* for 10 min. The supernatant was depigmented using a mixture of 1:3 (v/v) with chloroform, and the aqueous fraction was cold-dried for 12 h; the dried residue was resuspended in 500 ml of methanol with a reading of 520 nm, using sucrose as a standard. The determination of TSS was performed via spectrophotometry using the resorcinol method, expressed in mg/g of fresh weight (Roe, 1934).

The concentration of proline in foliar tissues was determined at each point of kinetics and in each treatment, following the slightly modified protocol of Bates et al. (1973). The foliar tissue was frozen with liquid nitrogen and pulverized in 3% sulfosalicylic acid. It was subsequently centrifuged at 16,000 *g* at room temperature for 20 min. An aliquot of the supernatant was added to 2 ml of ninhydrin reagent. The mixtures were kept in a water bath at 90°C for 1 h to develop the color. Once the tubes were cooled, 2 ml of toluene was added to separate the chromophore. For quantification, a 525 nm spectrophotometer was used, and the Proline values were expressed in umol/g^–1^ of fresh weight (for more details, see Molina-Montenegro et al., 2016).

Lipid peroxidation was estimated via the thiobarbituric acid (TBA) assay. The leaf tissue was homogenized in 0.1% trichloroacetic acid (TCA) and centrifuged at 10,000 *g* for 5 min; 1 ml of TBA (0.5%) in TCA (20%) was added to the aliquot of the supernatant and incubated at 90°C for 30 min; it was then left to cool to room temperature. The content of TBA reactive substances (TBARS) was determined at an absorbance of 532 nm and the non-specific absorbance of 600 nm; the lipoperoxidation values were expressed as mmol TBARS g^–1^ FW.

### Determination of Molecular Responses

#### Primer Design and cDNA Synthesis

Particles were designed for qPCR for the genes *CqNCED1*, *CqABCG25*, and *CqRD22*. The transcriptome of *C. quitensis* published by Bertini et al. (2015) was used to accomplish this. First, an amino acid database was made for each gene with the GenBank database^2^. Once the databases were generated, they were confronted against the transcriptome to select the contigs with a valid match: high percentage of coverage, identity, and negative e-value. The yield and purity of the RNA were verified by UV absorption spectra using NanoDrop Onem, a spectrophotometer UV–VIS spectral scan. RNA integrity was determined by electrophoresis in 2% agarose gel. The DNA was removed using the commercial TURBO DNA-Free Kit (Invitrogen). The cDNA was synthesized from the total RNA using the First Strand cDNA Synthesis commercial kit (Thermo Fisher Scientific, Waltham, MA, United States) according to the manufacturer’s instructions.

#### Analysis of Relative Expression of Genes for Synthesis, Transport, and Response to ABA

The expression of the genes was carried out by real-time PCR (RT-qPCR). To do this, a Stratagene Mx3000P thermal cycler was used (Agilent Technologies, United States). The amplification of the cDNA was done using the Maxima SYBR Green qPCR Master Mix (2X) kit (Thermo Scientific) in a final volume of 20 μl, containing 50 ng (2 μl) of cDNA, 10 μl of Maxima SYBR Green qPCR Master Mix (2X), 10 nM of forward and reverse for each gene, completing the final volume with nuclease-free water. For each biological replication, two technical replicas were made. The amplification profile used the following temperature: 95°C for 10 min, 40 cycles of 95°C × 30″, 60°C × 1′, 72°C × 20″ (Vashisth et al., 2011). A dissociation curve was generated at the end of each amplification program to verify that a single product was amplified, for which the temperature was increased from 55 to 95°C with a continuous fluorescence measurement. The calculations of the relative expression of each gene were made according to Pfaffl (2001) and analyses were made in relation to the normalizing gene GAPDH (Czechowski et al., 2005). The relative expression was recorded in 12 individuals per treatment and along a temporal kinetic at 0, 12, 36, 48, and 120 h.

### Determination of Functional Responses

#### Regulation of Stomatal Openings

To determine stomatal openings, the abaxial face of three leaves from five different individuals of *C. quitensis* with (E+) and without (E−) endophytes were analyzed upon being subjected to treatments of current water availability (W) and projected climate change (W+). The determination of stomatal openings was made at the end of the 5-day kinetics (120 h). The stomata were photographed and counted, taking three areas of 0.042 mm^2^ per leaf. The observation was made with an increase of 40X under the optical microscope (Zeiss Primo Star, ZEISS Germany). For the handling of images and quantification of the stomatal opening, the Motic Image Plus 3.0 software was used^3^.

#### Gas Exchange

The net photosynthesis rate and stomatal conductance were measured on a visually healthy leaf from 15 individuals corresponding to each treatment. Measurements were made on the same individual at midday and at 5 days, by an infrared gas analyzer (IRGA, Infra Red Gas Analyzer, CIRAS-2, PP-Systems, Haverhill, MA, United States).

### Data Analysis

Since in a preliminary analysis we do not found significant differences in factor “sampling site” for any assessed trait, data from different sites were merged in order to increase the sample size and in turn improve the explicative power of results obtained among treatments.

The effect of the presence of endophytic fungi in *C. quitensis* under the current condition (W−) and projected climate change condition (W+) on biochemical responses (proline, TSS, and TBARS) and relative expression of the *CqNCED1*, *CqABCG25*, and *CqRD22* was analyzed via two-way ANOVAs. We considered biochemical parameters and relative expression as response variables; the independent variables were time and the presence of endophytes. The analyzes were conducted independently for each environmental scenario (water levels), since the main objective of the study was to assess how the presence of endophytes affects osmoprotective mechanisms, rather than to compare different osmoprotective mechanisms among two different water conditions. In order to compare the effect of the presence of the endophytes on stomatal opening, net photosynthesis, and stomatal conductance, a two-way ANOVA was performed with endophytes (E− or E+) and water (W− or W+) as main factors. The differences between the treatments were evaluated by means of a Tukey test *a posteriori*. All analyzes were performed using STATISTICA 8.0.

## Results

### Determination of Biochemical Responses

#### Synthesis of Osmoprotective Molecules and Membrane Damage

**FIGURE 2:**
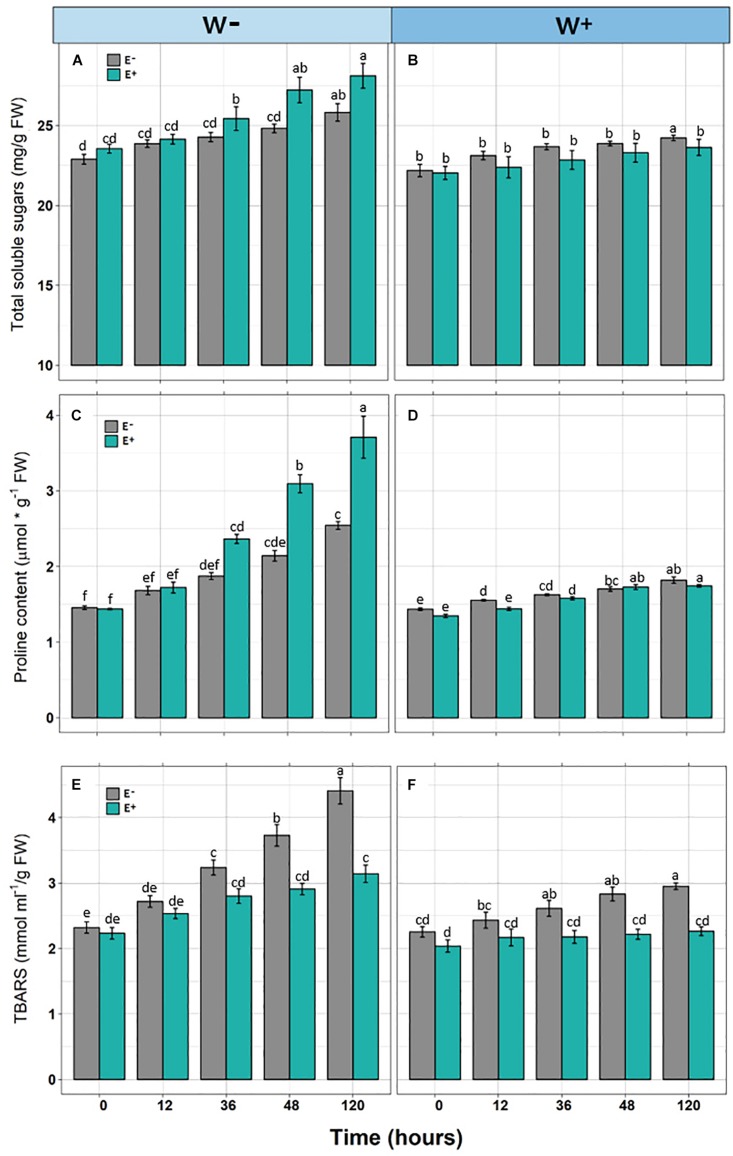
Biochemical responses [Total soluble sugars **(A,B)**, Proline content **(C,D)**, and TBARS **(E,F)**] in *Colobanthus quitensis* plants with (E+) and without (E−) endophytic fungi along experimental time under water condition currently found in Antarctica (W−) and the projected one according to climate change forecast (W+). Values are means ± SE. Different lowercase letters mean significant differences between treatments (Tukey test, *p* < 0.05).

In individuals E+and E−, and under the scenario of current water availability condition (W−) as well as the water availability condition in the climate change projection (W+), a significant increase in TSS levels was observed over time. However, they differ in the dynamics of increase, since TSS levels increased progressively with respect to zero time in E + W− individuals, whereas there was only a significant effect in the last point of kinetics in E−W individuals. Regarding the W+ condition, only at the last point of kinetics were significant differences found between E+ and E− individuals. Furthermore, since there was only one progressive effect of TSS increase in E+W− individuals, the overall interaction between time and presence of endophytes was not significant (Figures 2A,B and Table 1).

**TABLE 1:** Results of factorial ANOVA evaluating the presence of endophytes (E) and temporal kinetics for total soluble sugars (mg/g FW), proline (μmol/g FW), and TBARS (mmol mL^–1^/g FW) in plants of *Colobanthus quitensis* subjected to water content currently found in Antarctica (W−) and the projected one according to climate change forecast (W+).

	**Traits**	**df**	**MS**	***F***	***p***
**W−**	**Total soluble sugar**				
	Endophytes (E)	1	41.48	18.7	**0.0057**
	Time (T)	4	40.87	17.83	**0.0000**
	E × T	4	4.09	1.79	0.1398
	Error	80	2.29		
	**Proline**				
	Endophytes (E)	1	6.2357	60.828	**0.0000**
	Time (T)	4	8.3450	81.403	**0.0000**
	E × T	4	1.2659	12.348	**0.0000**
	Error	80	0.1025		
	**TBARS**				
	Endophytes (E)	1	7.0336	53.377	**0.0000**
	Time (T)	4	6.1529	46.693	**0.0000**
	E × T	4	1.0684	8.108	**0.0015**
	Error	80	0.1318		
**W+**	**Total soluble sugar**				
	Endophytes (E)	1	7.51	4.51	**0.0368**
	Time (T)	4	9.19	5.51	**0.0005**
	E × T	4	0.30	0.18	0.9477
	**Proline**				
	Endophytes (E)	1	0.0810	15.31	**0.001**
	Time (T)	4	0.4529	85.61	**0.0000**
	E × T	4	0.0129	2.43	0.0540
	Error	80	0.0053		
	**TBARS**				
	Endophytes (E)	1	4.4311	52.363	**0.0000**
	Time (T)	4	0.6001	7.091	**0.0006**
	E × T	4	0.1933	2.284	0.0674
	Error	80	0.0846		

*Significant effect factors (*P* < 0.05) are highlighted in bold.*

Similar to the levels of TSS, a significant progressive increase was observed with respect to the initial time in Proline levels in E+ individuals under the current condition of water availability (W−), compared to E− individuals. Although this pattern was observed in the W+ condition, proline levels were lower compared to condition W− (Figures 2C,D and Table 1).

Regarding TBARS, a significant progressive increase was observed in E− individuals, under the current condition of water availability (W−) and projected climate change (W+). In both conditions, E− individuals presented significantly higher levels compared to E+ individuals (Figures 2E,F). However, the interaction of time and presence of endophytes was only significant in condition W− (Figures 2E,F and Table 1).

### Determination of Molecular Responses

#### Relative Expression of Genes for Synthesis, Transport, and Response to ABA

**FIGURE 3:**
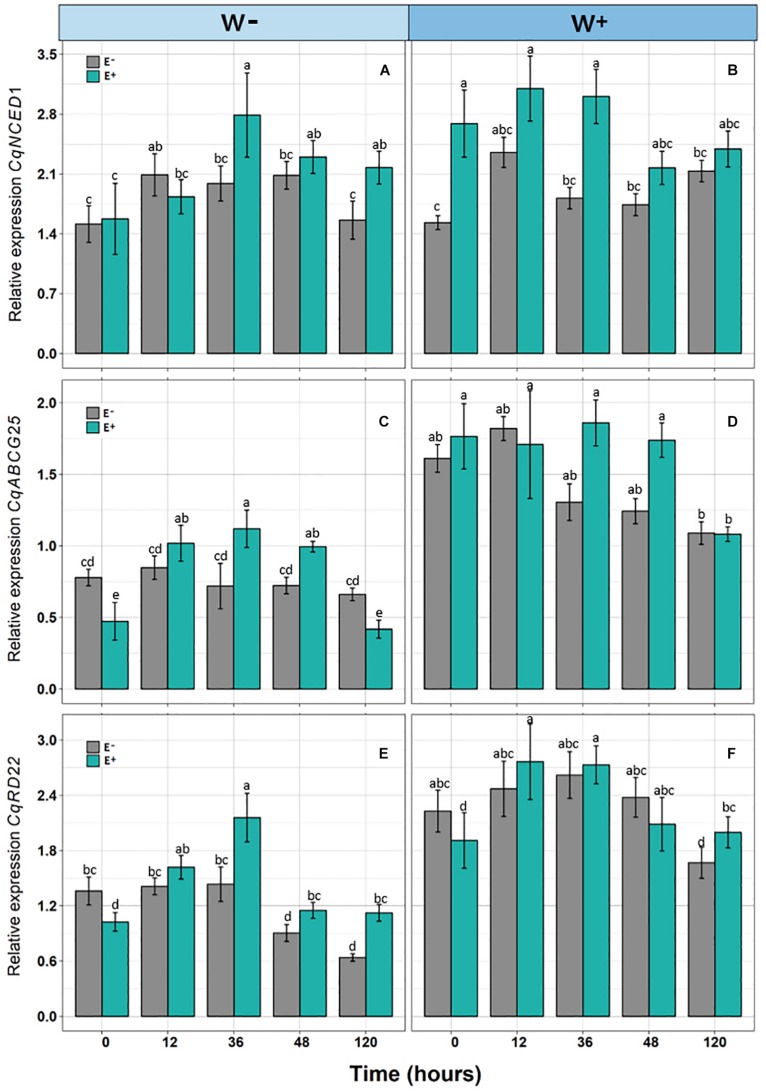
Relative expression of genes [*CqNCED1* **(A,B)**, *CqABCG25* **(C,D)**, and *CqRD22* **(E,F)**] in *Colobanthus quitensis* plants with (E+) and without (E−) endophytic fungi along experimental time, under water condition currently found in Antarctica (W−) and the projected one according to climate change forecast (W+). Values are means ± SE. Different lowercase letters mean significant differences between treatments (Tukey test, *p* < 0.05).

There was a significant positive effect found for the presence of endophytes (E+) on the relative expression of *CqNCED1* with respect to time 0, under the condition of current water availability (W−), and the transcription level was higher at 36 h. This pattern was not observed under the projected condition of climate change (W+); however, the number of transcripts was greater than condition W. Regarding the number of transcripts of *CqABCG25*, a significant effect of time was observed, as well as an interaction between time and presence of endophytes under the current condition of water availability (W−), and the largest number of transcripts was found at 36 h in individuals E + W. Regarding the projected condition of climate change (W+), time and presence of endophytes were only significant separately. Regarding the number of transcripts of *CqRD22* in the current condition of water availability (W−), a significant effect was observed in both time and the presence of endophytes, with the number of transcripts elevated at 36 h in individuals E + W−. In the W+ condition, only time had a significant effect on the number of transcripts (Figure 3 and Table 2).

**TABLE 2:** Results of factorial ANOVA evaluating the presence of endophytes (E) and temporal kinetics for relative level of transcripts by RT-qPCR of genes in *Colobanthus quitensis* subjected to water content currently found in Antarctica (W−) and the projected one according to climate change forecast (W+).

	**Traits**	**df**	**MS**	***F***	***p***
**W−**	***CqNCED1***				
	Endophytes (E)	1	14.4016	31.183	**0.0000**
	Time (T)	4	1.7873	3.870	**0.0353**
	E × T	4	0.9021	1.953	0.3620
	Error	80	0.4618		
	***CqABCG25***				
	Endophytes (E)	1	0.08921	0.9706	0.3269
	Time (T)	4	0.72968	7.9392	**0.0000**
	E × T	4	0.51276	5.5790	**0.0004**
	Error	80	0.09191		
	***CqRD22***				
	Endophytes (E)	1	1.7894	5.0450	**0.02701**
	Time (T)	4	2.5783	7.2694	**0.00003**
	E × T	4	0.8111	2.2867	0.06566
	Error	80	0.3547		
**W+**	***CqNCED1***				
	Endophytes (E)	1	2.1997	2.6039	**0.1097**
	Time (T)	4	2.2728	2.6904	**0.0059**
	E × T	4	0.9272	1.0975	0.1080
	Error	80	0.8448		
	***CqABCG25***				
	Endophytes (E)	1	1.3735	5.631	**0.01940**
	Time (T)	4	1.7482	7.167	**0.00003**
	E × T	4	0.5203	2.133	0.08152
	Error	80	0.2439		
	***CqRD22***				
	Endophytes (E)	1	0.0173	0.0240	0.87726
	Time (T)	4	2.8264	3.9279	**0.00532**
	E × T	4	0.5119	0.7113	0.58611
	Error	80	0.7196		

*Significant effect factors (*P* < 0.05) are highlighted in bold.*

### Determination of Functional Responses

#### Stomatal Opening

**FIGURE 4:**
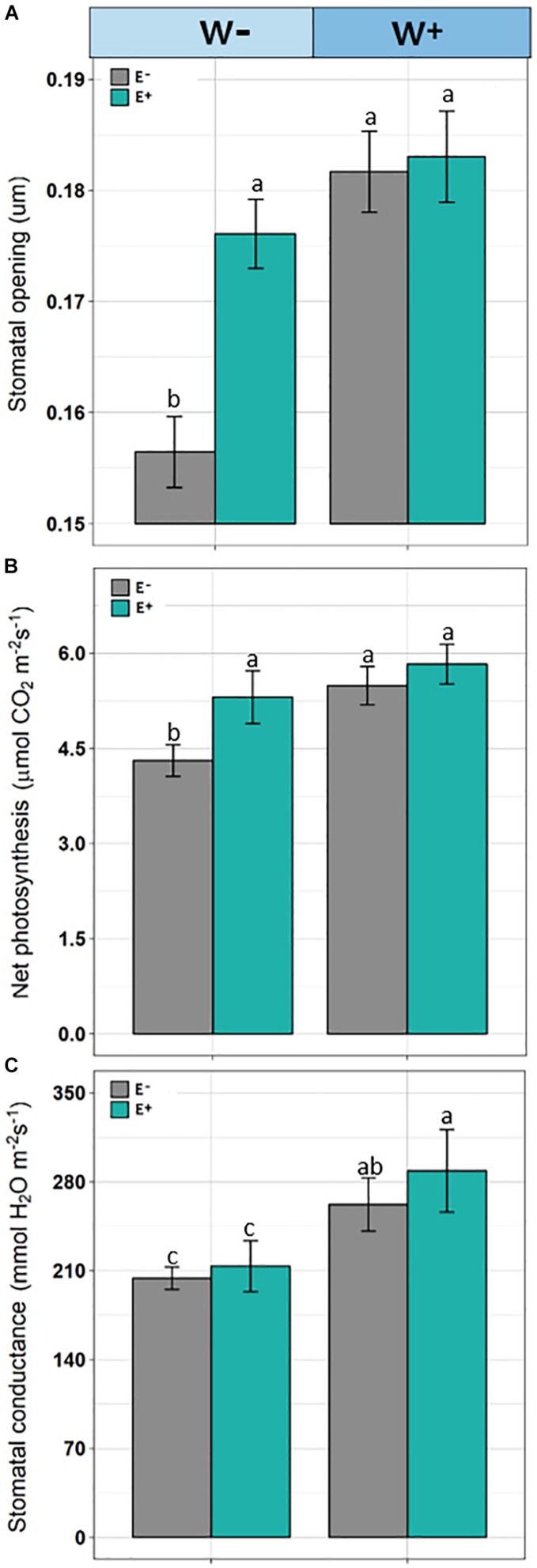
Stomata opening **(A)**, net photosynthesis **(B)**, and stomatal conductance **(C)** in plants of *Colobanthus quitensis* with (E+) and without (E−) endophytic fungi under water condition currently found in Antarctica (W−) and the projected one according to climate change forecasts (W+). Values are means ± SE. Different lowercase letters mean significant differences between treatments (Tukey test, *p* < 0.05).

Significant differences were observed (*F* = 8.290, *p* = 0.00024) in stomatal openings. Individuals E−W− presented a lower stomatal opening compared to E+ individuals under the current water availability condition (W−) and compared to both E+ and E− under the projected condition (Figure 4A).

#### Gas Exchange

Net photosynthetic rate was affected by root-endophyte presence and watering regime (*F* = 63.42; *p* < 0.001 and *F* = 102.08; *p* < 0.001, respectively). Net photosynthesis was significantly higher in the treatments with presence of root-endophytes and under projected water availability. Interaction between endophyte × water was significant, since under current water condition (W−), the net photosynthesis was decreased but with greater intensity in the absence of endophytes (*F* = 15.39; *p* = 0.002; Figure 4B). In addition, stomatal conductance was reduced in treatment without the presence of endophytes (*F* = 9.91; *p* = 0.0021) and under current water condition (F = 134.92; *p* < 0.001). Additionally, the interaction endophyte × water was significant (*F* = 15.39; *p* = 0.0018), because with decrease of water availability those individuals without presence of fungal endophytes showed lower stomatal conductance (Figure 4C).

## Discussion

Here, we showed how the presence of microorganisms contributes to enhance the drought tolerance in plants throughout expression of different osmoprotective mechanisms as well as differential gene expression (Timmusk et al., 2014; Ghaffari et al., 2016). Among the mechanisms induced by the presence of endophytes, there is a greater accumulation of solutes in tissues of vascular plants that allow osmotic adjustment and to maintain physiological and biochemical processes (Malinowski and Belesky, 2000; Li et al., 2018). This is consistent with the results observed here, where *C. quitensis* individuals grown under current conditions of water availability (W−), corresponding to water limitation, presented a significantly higher level of TSS and proline compared with E− individuals. Additionally, with E+ individuals presenting significantly lower values of TBARS compared to E− individuals, these results correlated with less damage from oxidative stress of membranes.

It has been shown that the presence of endophytes has a positive effect in some attributes of *C. quitensis* plants related to biological adaptation, which is more evident under conditions of water deficit (Torres-Díaz et al., 2016). Consistent with this, the differences in osmoprotective traits (TSS and proline) between E+ and E− plants clearly increased with the time and under water deficit. Nevertheless, these endophyte-driven differences were not observed under non-limiting water availability. On the other hand, lipoperoxidation was significantly higher in E− individuals in both water scenarios. Thus, fungal endophytes increase osmolytes like TSS and proline, which improve the membrane stability under drought condition, that in turn, is evidenced by low level of peroxidation of cell membranes. The presence of flavonoids and phenolic compounds has been noted in plant metabolomic profiles driven by endophytes when subjected to even low levels of abiotic stress, which eliminate hydroxyl groups, reducing the disintegration of hydroperoxides and inactivating lipid free radicals (Khan et al., 2016). The latter suggests that the presence of fungal endophytes contributes to improving the antioxidant potential in plants (Hamilton et al., 2012), and that the presence of endophytes is more relevant in activating this protective mechanism than the level of stress experienced by the plant. Nonetheless, if some osmoprotective mechanisms are independently of the endophyte presence, they would diminish the functional role of endophytes in a less limiting environmental scenario.

From a molecular perspective, this study is the first to evaluate genes associated with responses to water deficit in Antarctic plants. Overall, osmotic stress is considered as an environmental condition that quickly activates ABA biosynthesis (Xiong and Zhu, 2003). Once ABA has been synthesized, it is exported outside the cells through membrane transporters such as *ABCG25* (Park et al., 2017). Subsequently, it enters the guardian cells, and ABA signaling activates the outflow of potassium anions and ions through membrane transporters, with the subsequent decrease in pressure and turgor of the cells, which induces stomatal closure, avoiding the loss of water in the plant (Kim et al., 2010; Yoshida et al., 2019). Hence, the accumulation of the phytohormone ABA acts as the signaling mediator for regulating the adaptive response of plants to different environmental stress conditions as drought (Sah et al., 2016). Once ABA is synthesized, it must be exported to the intercellular space. The export can be performed through an ATP union cassette transporter *ABCG25*. It has been demonstrated in *Arabidopsis thaliana* plants, those individuals that overexpress *AtABCG25* showed higher leaf temperature, which implies an influence on stomatal regulation and closure (Kuromori et al., 2010). In the present study, a significant trend can be observed of increase in the relative expression of *CqNCED1*, *CqABCG25*, and *CqRD22*, with respect to zero time, in E+ individuals compared to E− individuals under current water availability conditions (W−). These results are consistent with other studies where the presence of the endophyte fungus *Piriformospora indica* in *A. thaliana* was associated to a higher and an earlier time expression in genes responding to dehydration in individuals subjected to drought stress (Sherameti et al., 2008). On the other hand, Antarctic fungal endophytes have been shown to improve the ecophysiological performance and fitness-related traits in several plant species exposed under different stress factors as water shortage (Molina-Montenegro et al., 2016), UV-B radiation (Ramos et al., 2018), and salinity (Acuña-Rodríguez et al., 2019). Therefore, these results along with those found in our study suggest the existence of a regulation at biochemical as well as transcriptional level, possibly mediated by the presence of the root endophytes, which would allow confer to different plant species—including *C. quitensis*—a greater tolerance to environmental constraints.

The current literature presents contradictory results regarding the effects of the endophyte symbiont in controlling the stomatal opening. On the one hand, it has been reported that they facilitate stomatal closure to conserve water, while other studies report that endophytes induce a greater stomatal opening to absorb more atmospheric CO_2_, thereby increasing photosynthesis and finally obtaining a greater biomass (Malinowski and Belesky, 2000; Rho et al., 2018). In the present study, *C. quitensis* plants presented a greater stomatal opening, increased net photosynthesis, and stomatal conductance as well as lower oxidative damage in the presence of root fungi. As suggested by Singh et al. (2016), the stomatal opening is controlled by a multifactorial signaling network in response to endogenous and environmental signals. Stomatal regulation in the leaves is associated with the level of ROS which, depending on the environmental conditions, acts as a signaling molecule for the movement of guard cells and the subsequent opening-closure of stomata.

Somehow in opposition to what was expected, levels of expression of the genes for synthesis, transport, and response to ABA under the projected condition of climate change (W+) were higher compared to those under current water condition (W−), which was more evident in E+ individuals than in E− individuals. This result could be partially explained by the susceptibility of the microbial community to micro-environmental changes, such as those driven by changes in soil water availability. For example, it was shown that the endophytic communities of bacteria in plants of *Oryza sativa* were significantly affected by variations in the physico-chemical soil conditions as a result of the flooding practices typically found in rice cropping systems (Ferrando and Fernández, 2015). Similarly, a study conducted with *Myricaria laxiflora* plants showed that flooding events negatively affected endophytic fungal communities, which were more diverse and abundant before flooding due to oxygen availability conditions (Tian et al., 2015). It has been suggested that plants are able to improve its responses to different biotic and/or abiotic conditions by retaining memories of stress factors, allowing a better performance under stochastic environmental stress events (Crisp et al., 2016). Although *C. quitensis* is distributed in areas with low water availability in the Antarctic, it can experience thawing events and/or sporadic liquid precipitation, which would explain the higher levels of gene expression endophyte-inoculated individuals. This differential expression may be due, on the one hand, to a synergistic response between the effects of increased water and presence of endophytes, producing stress in the microbial consortium. On the other hand, this may be due to the epigenetic memory of *C. quitensis* individuals that have been exposed to sporadic water stress (Hilker et al., 2016). However, it should be noted that this response was observed in the first three points of kinetics; afterward, the levels of expression decreased. Therefore, the potential response to stress due to excess water did not last over time, which is corroborated by the response in the physiological variables in the W+ condition, where the stress indicators were significantly lower compared to the current water condition (W−).

## Conclusion and Future Projections

Understanding the foundations by which fungal endophytes promote host plant adaptation to environmental conditions, imply to pass from factual to mechanistic experimental approaches. Thus, it is highly advisable to explore the impact of fungal endophytes on the host’s gene expression to establish the links with metabolism and physiology to finally impact on plant performance under different stressful conditions (Khare et al., 2018; Acuña-Rodríguez et al., 2019). Our results indicate that the presence of endophytes plays a positive role in the modulation of *C. quitensis* response to water stress at the biochemical (TSS, proline, and TBARS), morphological (stomatal opening), and molecular (*CqNCED1*, *CqABCG25*, and *CqRD22*) levels. This indicates that their interaction with endophytes is a successful strategy that induces a better response to water deficit in the plant. On the other hand, our results confirm that under the condition of climate change, the presence of endophytes would be less relevant, since minor differences were observed in the expression of mechanisms related with drought tolerance as well as growth and whole aspect between individuals E+ and E− compared to those individuals exposed to current water condition (see Figure 1 for this overall comparison). This is why it is interesting to evaluate the importance of symbiosis under future scenarios: to examine if, by reducing stress, the role of endophytes in the symbiotic relationship becomes unbalanced, turning from beneficial to neutral, and even evaluating whether the energy cost of the association outweighs the benefits for pathogenic effects on the plant. At the same time, the dynamics and changes in endophytic diversity modulated by long-term environmental conditions should be further studied, as well as how these changes could influence the transcriptional response of stress-response genes in *C. quitensis*, and the consequences on an ecological scale for the species in the Antarctic ecosystem.

## Data Availability Statement

The datasets analyzed in this manuscript are not publicly available. Requests to access the datasets should be directed to MM-M.

## Author Contributions

RH and MM-M designed the experiments. RH, SM-N, AB, and MM-M performed the experiments. RH, SM-N, GB, PR, and AB analyzed the data. MM-M wrote the manuscript along with RH and PG. All authors reviewed the manuscript.

## Conflict of Interest

The authors declare that the research was conducted in the absence of any commercial or financial relationships that could be construed as a potential conflict of interest.

## Footnotes

1 http://blast.ncbi.nlm.nih.gov/Blast.cgi

2 https://www.ncbi.nlm.nih.gov/genbank/

3 https://www.moticamseries.com/
